# Perceived artificial intelligence dependence and anxiety symptoms among university students: a three-wave study of experiential avoidance and digital literacy

**DOI:** 10.3389/fpsyt.2026.1934192

**Published:** 2026-10-19

**Authors:** Xu Liu, Zhenggui Gu, Lulu Shi

**Affiliations:** 1 School of Educational Sciences, Xinxiang University, Xinxiang, China; 2 Department of Human Development and Family Studies, Faculty of Human Ecology, Universiti Putra Malaysia, Serdang, Selangor, Malaysia; 3 School of Music, Xinxiang University, Xinxiang, China; 4 School of Education, Weifang University of Science and Technology, Weifang, China

**Keywords:** anxiety symptoms, artificial intelligence dependence, digital literacy, experiential avoidance, three-wave design, university students

## Abstract

University students increasingly use artificial intelligence tools for learning, decision-making, and academic work, making perceived AI dependence a relevant issue for public mental health and digital public health rather than only for educational technology. This three-wave time-lagged survey examined whether experiential avoidance functioned as a putative explanatory process linking perceived AI dependence with later anxiety symptoms and whether digital literacy moderated the tested pathways. Online survey data were collected from 1,451 university students with AI-use experience; perceived AI dependence, digital literacy, and baseline demographic variables were measured in January 2026, experiential avoidance was measured in March 2026, and anxiety symptoms were measured in June 2026. PLS-SEM results showed that experiential avoidance was strongly and positively associated with later anxiety symptoms (beta = 0.677, p < 0.001), whereas the direct association between perceived AI dependence and anxiety symptoms was not significant (beta = -0.030, p = 0.147); perceived AI dependence was negatively associated with experiential avoidance (beta = -0.114, p < 0.001), producing a statistically significant but theoretically unexpected negative time-ordered indirect association (beta = -0.077, p < 0.001), and digital literacy did not significantly moderate the tested pathways. The findings suggest that anxiety symptoms in AI-mediated learning contexts are closely linked to avoidance-related psychological processes, while the meaning of perceived AI dependence is context-dependent and should not be equated automatically with harmful use or clinical addiction; campus mental-health promotion should therefore integrate responsible AI education with support for psychological flexibility, uncertainty tolerance, and autonomous learning.

## 1 Introduction

University students are a population of public-health concern because anxiety symptoms are common during the transition to adulthood, academic competition, career uncertainty, and rapid changes in learning environments. A global systematic review and meta-analysis including 64 studies and 100,187 college students estimated the pooled prevalence of anxiety symptoms at 39.0% and depression symptoms at 33.6%, indicating that psychological distress among college students is not a marginal issue but a widespread challenge for higher education and public health (1–4). At the same time, higher education is entering an AI-intensive phase. AI applications have become increasingly embedded in teaching, learning analytics, feedback, writing, and student support systems, while systematic reviews note that the educational benefits of AI are accompanied by ethical, pedagogical, privacy, and risk-management concerns (5).

This makes AI dependence a public mental-health and digital public-health issue rather than merely an educational-technology issue. Digital technologies can shape students’ coping patterns, reassurance seeking, autonomy, and exposure to academic pressure; therefore, AI dependence should be examined as part of the broader ecology of digital well-being (6–8). In addition, research on automation has long shown that people may misuse, overuse, or defer excessively to automated systems, especially when they treat system outputs as authoritative without sufficient verification (9, 10). AI dependence is conceptually adjacent to automation overreliance and automation bias, but it is not identical to them: the present construct concerns students’ perceived psychological reliance on AI support, whereas automation-bias research focuses more directly on judgment errors and verification failure when automated advice is available. In university settings, similar overreliance on AI feedback may matter for mental-health promotion because it can affect how students manage uncertainty, competence threat, and academic decision-making.

The rapid diffusion of large language models and generative AI further intensifies this issue. UNESCO has emphasized that generative AI in education requires a human-centered approach, including privacy protection, pedagogical validation, and the development of human capacity rather than uncritical automation (11). Research on large language models in education likewise highlights their learning benefits but stresses the need for critical evaluation, fact-checking, and awareness of AI limitations (12, 13). Thus, the present study is not concerned simply with whether students use AI tools; it addresses whether perceived dependence on AI support is associated with anxiety-related psychological processes in a public mental-health context.

Existing research has developed several strands that are directly relevant to the present study. First, AI in higher education has been examined primarily from the perspectives of learning support, intelligent tutoring, academic writing, teaching innovation, ethics, and institutional governance (5, 11, 12). More recently, Morales-Garcia et al. developed and validated a five-item scale for dependence on artificial intelligence among university students, conceptualizing AI dependence as a psychological tendency involving insecurity without AI support, reassurance dependence on AI feedback, replacement anxiety, and competitive pressure (14). This scale provides a useful measurement basis for examining AI dependence as a psychological construct rather than merely as frequency of AI use.

Second, research on digital literacy suggests that students need more than access to digital tools; they also require knowledge, skills, and attitudes for locating information, evaluating content, communicating, creating, and acting critically in digital environments. Ng conceptualized digital literacy as a multidimensional competence relevant to digital societies, and the 10-item Digital Literacy Scale was subsequently adapted for student populations (15, 16). However, broad digital literacy should be distinguished from AI-specific literacy, which involves understanding AI capabilities and limitations, evaluating AI-generated outputs, recognizing automation bias, and maintaining responsible human judgment when algorithmic systems are available (9, 10, 17). This distinction is central to the present study because general digital competence may not be sufficient to buffer AI-specific psychological dependence.

Third, experiential avoidance provides a theoretically plausible putative psychological process linking AI-related dependence with anxiety symptoms. Experiential avoidance refers to unwillingness to remain in contact with unwanted internal experiences and attempts to avoid or control them, even when such avoidance creates longer-term psychological costs. The AAQ-II is widely used to assess experiential avoidance and psychological inflexibility (18), and the construct is grounded in acceptance and commitment therapy and psychological-flexibility theory (19, 20), although scholars have also cautioned against interpreting it too broadly without attention to discriminant validity (21). Empirical reviews and meta-analytic evidence support experiential avoidance as a transdiagnostic process associated with anxiety and related symptoms, with moderate-to-large associations across clinical and nonclinical domains (22, 23). Finally, anxiety symptoms are commonly assessed using the GAD-7, a brief and validated screening measure (24, 25). Together, these literatures suggest a plausible time-ordered pathway in which dependence on AI support may relate to anxiety symptoms through the way students respond to uncertainty, discomfort, and competence-related threat.

Despite these advances, several gaps remain. First, much of the higher-education AI literature focuses on adoption, academic integrity, pedagogical affordances, and ethical governance, whereas fewer studies examine AI dependence as a psychological risk factor for student mental health (5, 11, 14). Treating AI use and AI dependence as equivalent can obscure an important distinction: frequent AI use may reflect productive learning support, whereas dependence on AI reassurance may reflect insecurity, competence threat, or reduced confidence in independent decision-making.

Second, existing studies have not sufficiently clarified the putative psychological process through which AI dependence may be associated with later anxiety symptoms. Experiential avoidance is a theoretically relevant candidate because students who feel insecure without AI support may avoid uncertainty, difficult emotions, or independent problem-solving by seeking repeated AI reassurance. However, AI dependence, experiential avoidance, and anxiety symptoms have rarely been integrated into a single time-ordered model that separates antecedent, putative process, and outcome across measurement waves.

Third, the protective role of digital literacy remains theoretically plausible but empirically under-specified. Digital literacy is often treated as a general competence, yet it is unclear whether broad digital literacy is sufficient to weaken the links between AI dependence, avoidance processes, and anxiety symptoms. The digital well-being literature further suggests that healthy technology engagement depends not only on skill or access but also on how digital tools are integrated into everyday self-regulation, autonomy, and social functioning (8). Prior studies have not adequately tested whether digital literacy functions as a moderator in AI-related mental-health models, nor have they clearly distinguished general digital competence from AI-specific literacy skills.

Fourth, prior research is often cross-sectional, limiting the temporal interpretation of indirect-effect models. A model that measures predictor, putative process, and outcome at the same time cannot clearly establish whether AI dependence precedes experiential avoidance or whether anxiety symptoms influence both. A three-wave time-lagged design is therefore useful for establishing a basic temporal order before statistical testing, even though it does not by itself prove strict causality or within-person change.

To address these gaps, the present study examined the association between AI dependence and later anxiety symptoms among university students. The study adopted a three-wave time-lagged design: AI dependence, digital literacy, and demographic variables were measured at T1 in January 2026; experiential avoidance was measured at T2 in March 2026; and anxiety symptoms were measured at T3 in June 2026. The analytic model tested whether experiential avoidance was involved in a time-ordered indirect association between AI dependence and subsequent anxiety symptoms and whether digital literacy functioned as a moderator in the model. The study also evaluated measurement properties, structural paths, indirect associations, interaction terms, explained variance, and predictive relevance using PLS-SEM.

Given the current state of the literature and the limits of the design, the study was framed as an investigation of time-ordered associations rather than as a test of deterministic causal effects. This wording is important because the design separates the key constructs across time but does not repeatedly measure all focal variables at every wave. Thus, the objective was to clarify whether AI dependence, experiential avoidance, digital literacy, and anxiety symptoms are systematically connected in a theoretically meaningful sequence, while keeping interpretation proportional to the design.

Based on the literature and the time-lagged framework, the study tested H1 (T1 perceived AI dependence → T3 anxiety symptoms), H2 (T1 perceived AI dependence → T2 experiential avoidance), H3 (T2 experiential avoidance → T3 anxiety symptoms), H4 (the model-based indirect association AID → EA → ANX), and H5a-H5c (digital-literacy moderation of AID → EA, AID → ANX, and EA → ANX, respectively). **Figure 1** uses this same numbering and pathway structure.

**Figure 1:**
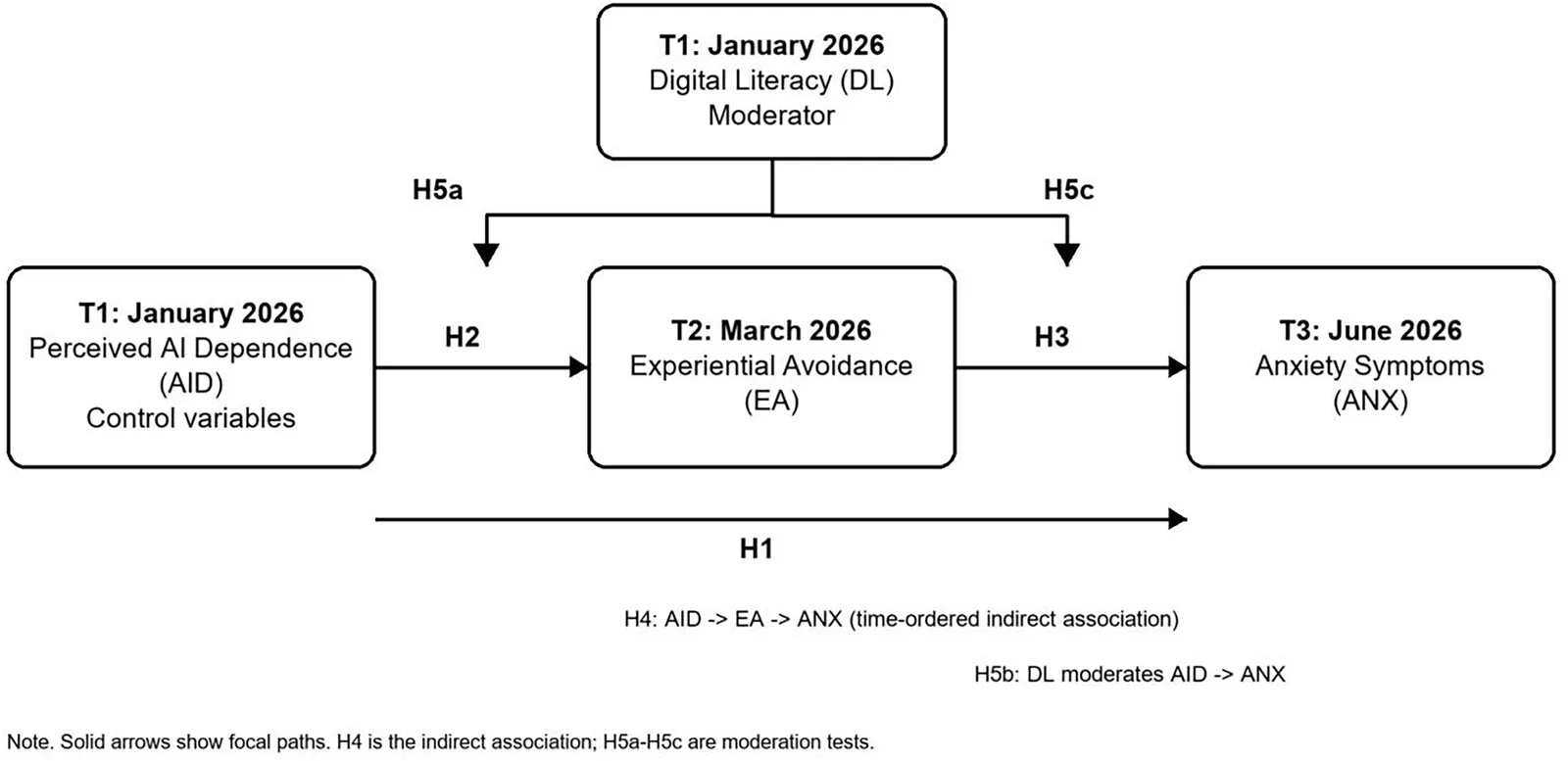
Simplified three-wave conceptual model.

Because AI dependence may reflect both maladaptive reassurance seeking and active AI-supported learning, the direction of the AI dependence–experiential avoidance association was treated as an empirical question rather than assumed to be uniformly risk-enhancing.

This study makes several contributions. Theoretically, it extends student mental-health research into the AI-mediated learning context by treating AI dependence as a psychological construct rather than merely as technology exposure or usage frequency. This responds to the need for more psychologically grounded accounts of how AI may affect students beyond academic performance and academic integrity. It also treats experiential avoidance as a putative process rather than assuming a confirmed mechanism, allowing the empirical direction of the indirect association to challenge a simple risk-escalation pathway.

Methodologically, the study improves on purely cross-sectional designs by using a three-wave time-lagged structure that separates the predictor, mediator, and outcome in time. This strengthens temporal ordering and reduces same-time measurement bias, while still acknowledging the limits of causal inference. Practically, the findings may inform university mental-health promotion and AI-literacy education by highlighting that responsible AI use should include not only technical skills but also psychological self-regulation, critical evaluation of AI feedback, and awareness of overreliance on automated support (8–10).

## 2 Materials and methods

**Figure 1** presents the three-wave conceptual model with H1-H3 labeling the focal direct paths, H4 labeling the AID → EA → ANX model-based indirect association, and H5a-H5c labeling the three digital-literacy moderation tests.

### 2.1 Study design and conceptual framework

This study adopted a three-wave time-lagged design to examine the association between perceived artificial intelligence dependence and subsequent anxiety symptoms among university students in the digital era. The conceptual framework positioned perceived artificial intelligence dependence (AID) as the antecedent variable, experiential avoidance (EA) as a time-ordered psychological process, and anxiety symptoms as the focal mental-health outcome. Digital literacy (DL) was conceptualized as a moderator and digital competence factor. The model therefore tested direct paths from AID to EA and anxiety symptoms, the indirect path from AID to anxiety symptoms through EA, and moderation paths involving DL.

The three waves were arranged to establish a basic temporal order before statistical testing. At Time 1 in January 2026, AID, DL, and demographic control variables were measured. At Time 2 in March 2026, EA was measured as the putative explanatory process. At Time 3 in June 2026, anxiety symptoms were measured as the outcome. This design reduces same-time measurement bias and makes the indirect-association logic more consistent with a time-ordered sequence from AI-related behavior to psychological process and later anxiety symptoms. However, because all core constructs were not repeatedly measured at each wave, the design should be interpreted as time-lagged and associational rather than as evidence of strict causal inference or within-person change.

### 2.2 Data source, sampling, and participants

Data were collected in Chinese through Wenjuanxing at two universities in China: one urban institution and one suburban institution. Purposive recruitment covered first-to fourth-year students in liberal-arts and science-related programs who had prior AI-use experience. The two sites were not named to protect institutional confidentiality. At each wave, the online questionnaire was administered during a scheduled face-to-face class session, and students completed the survey on site using Wenjuanxing. Students were reminded to participate before each wave. To encourage continued participation, students who completed all three waves received a two-point addition to their continuous-assessment grade. Students who chose not to participate in the study or did not complete all three survey waves could earn the same two continuous-assessment marks through alternative classroom tasks. Accordingly, declining or discontinuing research participation did not academically disadvantage students. These class-based, on-site administration and follow-up procedures contributed to the high retention rate. The number reported as ‘invited at T1’ refers to students who entered and completed the baseline survey, not to all students who may have seen the invitation; therefore, a population-level participation rate cannot be calculated from the available records.

The online questionnaire required a response to every item, so no item-level missing values were present in the retained records. No device restriction was imposed. Response-time screening used a plausible completion pace of approximately 2–8 seconds per item; no additional records were excluded solely because of device type or response time after matching and duplicate screening. According to the survey records, 1,455 students completed T1, 1,453 completed T2, and 1,453 completed T3. Two repeated student-ID records were identified; the first valid record was retained in each case, leaving 1,451 valid matched cases for analysis.

Attrition across waves was minimal: two baseline participants were not retained at follow-up. Retention was supported by three procedures: administration of each online survey during a scheduled face-to-face class session, a reminder before every wave, and a two-point continuous-assessment incentive for students who completed the entire three-wave study. The student ID used for longitudinal linkage was the official ID assigned at university registration. It was used solely to match records across waves and identify duplicate submissions. Student IDs were kept confidential throughout data collection, removed before analysis, and excluded from the analytic dataset; no participant names were included in the analysis. Because recruitment was purposive and restricted to students with AI-use experience, the sample is not statistically representative; AI-use frequency was not measured, so subgroup comparisons by low, moderate, or high use could not be performed.

**Table 1** summarizes the demographic composition of the analytic sample (N = 1,451), including age, academic achievement (CGPA), gender, family income, and parental education. The wave-specific completion and duplicate-screening counts are reported above.

**Table 1:** Sample characteristics of the analytic sample.

Characteristic	Statistic
Final analytic sample	1,451 valid matched cases after excluding two duplicate records
Gender	437 male; 1,014 female
Age, years	M = 19.51, SD = 1.19, range = 18-24
CGPA	M = 3.31, SD = 0.42, range = 1.00-4.00
Family monthly income	M = 8,558.29, SD = 5,295.90, range = 2,000-40,000
Maternal education, years	M = 10.32, SD = 2.41, range = 6-20
Paternal education, years	M = 10.59, SD = 2.25, range = 9-20

M, mean; SD, standard deviation.

### 2.3 Ethics, informed consent, and privacy protection

This study was conducted at two universities in China in accordance with the Declaration of Helsinki (26). The Ethics Committee for Human Subjects Research of Universiti Putra Malaysia approved the protocol (Approval No. JKEUPM-2023-1426) within the lead researcher’s institutional research framework. The participating Chinese universities provided site-level administrative authorization; no additional local ethics-committee approval is recorded in the study documentation. Electronic written informed consent was required and documented at baseline before students entered the three-wave follow-up, and participation remained voluntary at every wave. Student IDs were used only for longitudinal linkage and duplicate screening and were deleted before analysis; only de-identified data were analyzed.

### 2.4 Measures

All four instruments were administered in Chinese. The AAQ-II and GAD-7 used established Chinese-language versions. Because validated Chinese versions of the five-item Dependence on Artificial Intelligence Scale and the 10-item Digital Literacy Scale were not available for this study, those instruments were translated independently by two bilingual researchers, reconciled, back-translated, reviewed by subject-matter experts, and pilot-tested for semantic and contextual equivalence. Permission to use and translate the instruments was obtained from the relevant developers. All measures were self-reports and were not interpreted as diagnostic classifications (**Table 2**).

**Table 2:** Four-measure summary.

Measure	Wave	Chinese-language status	Scoring
Perceived AI dependence	T1	Translated and back-translated for this study	5-point mean; higher = greater perceived reliance
Digital literacy	T1	Translated and back-translated for this study	5-point mean; higher = greater general digital literacy
AAQ-II	T2	Established Chinese version	7-point mean; higher = greater experiential avoidance
GAD-7	T3	Established Chinese version	4-point mean; higher = greater anxiety symptoms

#### 2.4.1 Artificial intelligence dependence

AI dependence was measured at T1 using the Chinese version of the five-item Dependence on Artificial Intelligence Scale developed and validated among university students by Morales-Garcia et al. (14). The scale was designed to assess students’ subjective dependence on artificial intelligence in learning, decision-making, and academic activities. The items capture two related content domains: AI-related insecurity and reassurance dependence, and AI-driven competence threat. Participants responded on a 5-point Likert scale, and item scores were averaged, with higher scores indicating greater perceived AI dependence. The scale should be interpreted as perceived psychological dependence on AI support rather than as an objective measure of AI-use frequency, pathological technology addiction, or clinical impairment.

#### 2.4.2 Digital literacy

Digital literacy was measured at T1 using the Chinese version of the 10-item Digital Literacy Scale originally developed by Ng (15) and adapted by Ustundag et al. (16). The scale assesses students’ perceived knowledge, skills, and attitudes for locating, evaluating, communicating, creating, and acting critically in digital environments. Participants responded on a 5-point Likert scale, and item scores were averaged, with higher scores indicating higher general digital literacy. In the structural model, digital literacy was specified both as a focal competence variable and as the moderator of the hypothesized AI-dependence-related and experiential-avoidance-related pathways. Because this instrument captures broad digital competence rather than AI-specific literacy, moderation results were interpreted cautiously.

#### 2.4.3 Experiential avoidance

EA was measured at T2 using the Chinese version of the seven-item Acceptance and Action Questionnaire-II (AAQ-II), a widely used measure of psychological inflexibility and experiential avoidance (18). Authorization to use the scale was obtained from the scale developer. Items were scored on a 7-point response scale and averaged, with higher scores indicating greater experiential avoidance. Because subsequent work has raised concerns about the discriminant validity of the AAQ-II when it is interpreted too broadly (21), the present study treated the scale specifically as an index of experiential avoidance/psychological inflexibility rather than as a stand-alone diagnostic measure. EA was specified as a putative time-ordered explanatory process linking T1 AID with T3 anxiety symptoms.

#### 2.4.4 Anxiety symptoms

Anxiety symptoms were measured at T3 using the Chinese version of the seven-item Generalized Anxiety Disorder scale (GAD-7), a brief self-report measure developed to assess generalized anxiety symptoms (24) and subsequently validated in general-population settings (25). Authorization to use the scale was obtained from the scale developer. Items were scored on a 4-point response scale and averaged, with higher scores indicating more severe anxiety symptoms. In the present study, anxiety was treated as a continuous symptom indicator and screening construct rather than as a clinical diagnosis.

#### 2.4.5 Control variables

Baseline demographic variables measured at T1 included gender, age, family monthly income, maternal years of education, and paternal years of education. Gender was treated as a categorical variable, whereas age, family monthly income, and parental education years were treated as continuous variables. These variables were collected to describe baseline demographic heterogeneity and were additionally used in a sensitivity analysis. In that sensitivity model, the focal structural equations were re-estimated using standardized composite-score regressions, with gender, age, family income, maternal education, and paternal education included as covariates predicting experiential avoidance and anxiety symptoms.

### 2.5 Data screening and preliminary analyses

Before testing the structural model, the three-wave data were matched using student IDs and then de-identified by deleting the matching codes. The participant-flow records showed that 1,455 students completed T1, 1,453 completed T2, 1,453 completed T3, and 1,453 records were matched across the three waves. Two duplicate student-ID records were excluded, with the first valid record retained in each case, leaving 1,451 final analytic cases. The retained analytic dataset contained complete data for the focal item-level indicators and baseline demographic variables because every online questionnaire item was mandatory.

Data screening examined duplicate records, plausible completion time, permissible response ranges, scoring direction, item-total direction, and descriptive distributions. All AID, DL, AAQ-II, and GAD-7 items were keyed so that higher values represented more of the named construct; no reverse-scored items, miscoded responses, or response-anchor inversions were identified. Recalculation from the item-level data reproduced the reported composite-score directions. The five-item AID scale was analyzed as the unidimensional instrument validated by its developers, so *post hoc* dimensional analyses were not conducted.

Full collinearity VIFs were additionally examined following the recommended PLS-SEM diagnostic approach (27), and all values were below 3.3, suggesting that method bias was unlikely to dominate the observed associations.

### 2.6 PLS-SEM analytic strategy

PLS-SEM was selected *a priori* because the study tested a comparatively new structural model centered on perceived AI dependence, three product-indicator interactions, and specific indirect associations, and because the analytic objectives included estimating explained variance, small interaction effects, and out-of-sample prediction diagnostics within one framework (28). This choice was not based on distributional problems or sample-size limitations. Covariance-based SEM would be appropriate for a primarily confirmatory latent-variable model emphasizing global fit, but the present analysis prioritized variance explanation, prediction diagnostics, and interaction estimation. The analysis was conducted in SmartPLS 4 (29). The positive but small Q²_predict values ultimately showed limited predictive relevance; accordingly, PLS-SEM is treated here as an estimation strategy rather than evidence that the model has strong predictive performance. Fully repeated measures were unavailable, so cross-lagged or latent-change SEM could not be validly estimated.

For the measurement model, indicator loadings were inspected, and internal consistency was assessed using Cronbach’s alpha, rho-A, and composite reliability. Convergent validity was evaluated using the average variance extracted (AVE), consistent with established measurement-model evaluation criteria (30, 31). Discriminant validity was examined using the heterotrait-monotrait ratio (HTMT), a recommended criterion in variance-based SEM (32). Collinearity was evaluated using variance inflation factors. For the structural model, standardized path coefficients, standard errors, t statistics, p values, and 95% confidence intervals were obtained from bootstrapping. Bootstrapping was performed with 5,000 subsamples using a two-tailed test, a 0.05 significance level, a fixed random-number seed, and percentile bootstrap 95% confidence intervals. Moderation effects were estimated using the product-indicator approach in SmartPLS 4. PLSpredict was conducted with 10 folds and 10 repetitions, using the linear model benchmark for RMSE and MAE comparisons; the PLSpredict PLS setup used path weighting, standardized results, and the default initial-weight setting. The model further reported R2 for endogenous constructs, f2 effect sizes, Q2_predict values, and specific indirect effects; predictive relevance was interpreted in line with PLSpredict guidance (33). As a sensitivity analysis, the focal structural equations were re-estimated using standardized composite-score regressions, with gender, age, family income, maternal education, and paternal education included as covariates predicting experiential avoidance and anxiety symptoms. The focal path estimates remained substantively unchanged, suggesting that the main conclusions were not driven by baseline demographic differences. The unadjusted and covariate-adjusted sensitivity estimates are reported in **Supplementary Table S1**.

### 2.7 Interpretation discipline

Because the design was time-lagged rather than a fully repeated panel, all paths were interpreted as time-lagged associations, the indirect result as a model-based indirect association, and experiential avoidance as a putative explanatory process. No claim of confirmed psychological mediation, within-person change, or causality is made. The negative AID-to-EA path was retained only after the scoring audit found no reversed items, miscoding, or anchor inversion.

## 3 Results

### 3.1 Preliminary analyses

Because the main variables were assessed using self-report instruments, common method variance was examined before testing the structural model. The three-wave design separated the measurement of the predictor and moderator variables, the mediator, and the outcome. Harman’s single-factor test was conducted using all 29 observed indicators of AI dependence, experiential avoidance, digital literacy, and anxiety symptoms. The first unrotated factor accounted for 30.63% of the total variance, below the conventional 50% threshold, suggesting that common method variance was unlikely to be the dominant source of covariance among the study variables (34). However, because Harman’s single-factor test is only a diagnostic screening procedure, common method bias cannot be ruled out. Full collinearity VIFs were additionally examined, and all values were below 3.3, suggesting that method bias was unlikely to dominate the observed associations.

Descriptive statistics and bivariate correlations are shown in **Table 3**. AID was negatively correlated with EA and ANX, but positively correlated with DL. EA was strongly and positively correlated with ANX and weakly negatively correlated with DL. DL was weakly and negatively correlated with ANX. These preliminary associations indicate a central association between EA and ANX, whereas the correlations involving AID and DL were small in magnitude.

**Table 3:** Descriptive statistics and correlations among study variables.

Variable	M	SD	alpha	1	2	3	4
1. AID	2.63	0.71	.845	–			
2. EA	3.15	1.33	.939	-.112***	–		
3. DL	4.08	0.61	.933	.064*	-.098***	–	
4. ANX	1.75	0.63	.937	-.105***	.680***	-.080**	–

N = 1,451. alpha = Cronbach’s alpha. AID and DL were measured on 5-point scales, EA on a 7-point scale, and ANX on a 4-point scale. *p < 0.05; **p < 0.01; ***p < 0.001. AID, artificial intelligence dependence; EA, experiential avoidance; DL, digital literacy; ANX, anxiety symptoms.

### 3.2 Measurement model

The measurement model demonstrated acceptable reliability and convergent validity (**Table 4**). Cronbach alpha values ranged from 0.845 to 0.939, composite reliability values ranged from 0.885 to 0.951, and AVE values ranged from 0.608 to 0.733. These results met commonly used criteria for internal consistency and convergent validity (30, 31). Discriminant validity was assessed using HTMT (**Table 5**), and all reported HTMT values were below conventional cutoffs, with the highest value being 0.725 for EA and ANX (32). Inner model collinearity was also acceptable, with VIF values ranging from 1.006 to 1.048 (**Table 6**).

**Table 4:** Indicator loadings, reliability, and convergent validity for focal constructs.

Construct	Item	Loading	Cronbach alpha	rho_A	rho_C	AVE
AID	AID1	.783	.845	.888	.885	.608
AID	AID2	.849				
AID	AID3	.742				
AID	AID4	.805				
AID	AID5	.748				
DL	DL1	.775	.933	.962	.942	.621
DL	DL2	.790				
DL	DL3	.811				
DL	DL4	.777				
DL	DL5	.835				
DL	DL6	.739				
DL	DL7	.848				
DL	DL8	.709				
DL	DL9	.819				
DL	DL10	.785				
EA	EA1	.856	.939	.941	.951	.733
EA	EA2	.869				
EA	EA3	.887				
EA	EA4	.809				
EA	EA5	.868				
EA	EA6	.818				
EA	EA7	.885				
ANX	ANX1	.838	.937	.939	.949	.728
ANX	ANX2	.888				
ANX	ANX3	.901				
ANX	ANX4	.877				
ANX	ANX5	.829				
ANX	ANX6	.808				
ANX	ANX7	.828				

Loadings are standardized item-construct loadings. Reliability and validity statistics are reported once for each construct. rho_A = Dijkstra-Henseler reliability coefficient; rho_C = composite reliability; AVE, average variance extracted. AID, artificial intelligence dependence; DL, digital literacy; EA, experiential avoidance; ANX, anxiety symptoms.

**Table 5:** Discriminant validity: HTMT matrix.

Construct	1	2	3	4	5	6
1. AID	–					
2. ANX	.116	–				
3. DL	.074	.090	–			
4. EA	.123	.725	.103	–		
5. DL × EA	.015	.076	.076	.119	–	
6. DL × AID	.009	.019	.137	.011	.161	–

Values are heterotrait-monotrait ratios. Lower triangle is shown. AID, artificial intelligence dependence; EA, experiential avoidance; DL, digital literacy; ANX, anxiety symptoms.

**Table 6:** Structural model path coefficients, inner model collinearity statistics, and effect sizes.

Path	β	M	SE	t	p	95% CI	VIF	f²
AID → ANX	−.030	−.031	.021	1.451	.147	[−.072,.009]	1.020	.002
AID → EA	−.114	−.117	.025	4.518	<.001	[−.166, −.069]	1.006	.013
DL → ANX	−.011	−.011	.023	0.494	.621	[−.054,.034]	1.040	.000
DL → EA	−.100	−.106	.028	3.613	<.001	[−.164, −.058]	1.023	.010
DL × AID → ANX	.019	.017	.020	0.966	.334	[−.021,.057]	1.048	.001
DL × AID → EA	−.021	−.022	.031	0.677	.498	[−.084,.040]	1.017	.001
DL × EA → ANX	−.002	−.001	.025	0.078	.937	[−.046,.052]	1.048	.000
EA → ANX	.677	.676	.018	37.411	<.001	[.641,.711]	1.037	.827

beta, original sample estimate; M, bootstrap sample mean; SE, bootstrap standard error; CI, confidence interval; VIF, variance inflation factor; f2, Cohen effect size for the structural path. AID, artificial intelligence dependence; EA, experiential avoidance; DL, digital literacy; ANX, anxiety symptoms.

### 3.3 Structural model and hypothesis tests

The structural model results are presented in **Table 6** and are interpreted against the hypotheses and research questions. H1 was not supported because the direct path from AI dependence to anxiety symptoms was not statistically significant. H2 was statistically significant but opposite to a simple risk-escalation expectation, as AI dependence was negatively associated with experiential avoidance (beta = -0.114, p < 0.001). H3 was supported, with experiential avoidance positively associated with anxiety symptoms (beta = 0.677, p < 0.001). H4 showed a statistically significant but theoretically unexpected negative time-ordered indirect association from AI dependence to anxiety symptoms through experiential avoidance (beta = -0.077, p < 0.001). H5 was not supported because the digital-literacy interaction terms were not statistically significant. Digital literacy was negatively associated with experiential avoidance (beta = -0.100, p < 0.001), but its direct association with anxiety symptoms was not statistically significant. The hypothesis decisions are summarized in **Table 7**.

**Table 7:** Hypothesis decision summary.

Hypothesis	Tested path/effect	Result	Decision
H1	AID → ANX	beta = -.030, p = .147	Not supported
H2	AID → EA	beta = -.114, p <.001	Association supported; negative direction
H3	EA → ANX	beta = .677, p <.001	Supported
H4	AID → EA → ANX	beta = -.077, p <.001	Supported as a negative time-ordered indirect association
H5a	DL × AID → EA	beta = -.021, p = .498	Not supported
H5b	DL × AID → ANX	beta = .019, p = .334	Not supported
H5c	DL × EA → ANX	beta = -.002, p = .937	Not supported

ANX, anxiety symptoms; AID, artificial intelligence dependence; DL, digital literacy; EA, experiential avoidance.

Specific indirect effects are presented in **Table 8**. The conditional indirect effect involving the digital literacy × AI dependence interaction was not significant.

**Table 8:** Specific indirect effects.

Indirect path	beta	M	SE	t	p	95% CI
DL × AID → EA → ANX	-.014	-.015	.021	0.680	.497	[-.056,.027]
AID → EA → ANX	-.077	-.079	.017	4.528	<.001	[-.113, -.047]
DL → EA → ANX	-.068	-.072	.019	3.577	<.001	[-.111, -.039]

beta = original sample estimate; M = bootstrap sample mean; SE = bootstrap standard error; CI = confidence interval. AID, artificial intelligence dependence; EA, experiential avoidance; DL, digital literacy; ANX, anxiety symptoms.

The model explained 46.6% of the variance in anxiety symptoms and 2.5% of the variance in experiential avoidance (**Table 9**). The low explained variance for experiential avoidance indicates that AI dependence, digital literacy, and their interaction accounted for only a small portion of between-person differences in experiential avoidance; therefore, the antecedent side of the model should be interpreted cautiously. As shown in **Table 6**, the strongest effect size was observed for experiential avoidance → anxiety symptoms (f^2^ = 0.827), whereas the remaining structural paths showed very small effect sizes. Predictive relevance was evaluated using Q2_predict, RMSE, and MAE in line with PLS-SEM prediction guidance (33). Q2_predict values were positive but small, indicating limited predictive relevance; therefore, the findings should be interpreted primarily as evidence of structural associations rather than strong out-of-sample prediction. In the covariate-adjusted composite-score sensitivity model, adding gender, age, family income, maternal education, and paternal education as predictors of experiential avoidance and anxiety symptoms did not materially change the focal path pattern (**Supplementary Table S1**).

**Table 9:** Explained variance and predictive relevance.

Endogenous construct	R2	Adjusted R2	Q2_predict	RMSE	MAE
ANX	.466	.464	.010	.996	.758
EA	.025	.023	.017	.993	.799

RMSE, root mean square error; MAE, mean absolute error. AID, artificial intelligence dependence; EA, experiential avoidance; DL, digital literacy; ANX, anxiety symptoms.

## 4 Discussion

### 4.1 Principal findings

This study examined how artificial intelligence dependence was linked to later anxiety symptoms among university students and whether experiential avoidance and digital literacy helped clarify this relationship. The most stable finding was that experiential avoidance was strongly and positively associated with subsequent anxiety symptoms. This result is consistent with the broader psychological literature showing that experiential avoidance and psychological inflexibility are central processes in anxiety and emotional distress (18, 21, 22). In contrast, the direct effects of AI dependence and digital literacy on anxiety symptoms were not significant, and the hypothesized moderation effects involving digital literacy were not supported. The indirect associations through experiential avoidance were statistically significant but negative. Therefore, the findings do not support a simple risk-escalation story in which AI dependence directly increases anxiety or does so by increasing experiential avoidance. Instead, the results point to a more complex psychological pattern: anxiety symptoms were closely tied to avoidance processes, whereas AI dependence and digital literacy showed smaller, more context-dependent associations with that putative explanatory process. The positive but small Q2_predict values indicate limited predictive relevance, so the structural paths should be interpreted more as explanatory associations than as strong out-of-sample prediction.

This pattern is important because it prevents an overly deterministic conclusion about AI use in higher education. Prior work has emphasized both the opportunities and risks of AI in higher education, including learning support, intelligent tutoring, academic writing, institutional governance, and ethical concerns (5, 11, 12). The present findings suggest that the mental-health implications of AI use may depend less on the mere presence of AI tools and more on how students psychologically relate to those tools. AI dependence may reflect insecurity and reassurance seeking for some students, but it may also coexist with active engagement, academic problem solving, or instrumental use for others. This distinction is consistent with the AI dependence scale itself, which measures perceived psychological dependence rather than objective frequency of AI use (14).

### 4.2 Why experiential avoidance was strongly associated with anxiety symptoms

The strong association between experiential avoidance and anxiety symptoms can be understood through the logic of psychological inflexibility. Experiential avoidance involves attempts to escape, suppress, or control unwanted internal experiences. In academic contexts, students may experience uncertainty, fear of poor performance, comparison pressure, and worry about competence. If students respond to these internal experiences by avoidance rather than engagement, short-term relief may come at the cost of longer-term anxiety. This mechanism is consistent with the AAQ-II literature, in which experiential avoidance is treated as a core component of psychological inflexibility (18), and with meta-analytic evidence showing robust associations between experiential avoidance and anxiety-related symptoms (22).

The result also fits the public-health framing of the study. Anxiety symptoms among university students are highly prevalent, with meta-analytic evidence showing substantial global levels of anxiety symptoms in college populations (1). The current study suggests that, in an AI-mediated learning environment, a transdiagnostic psychological process remains central: students who are less willing or able to tolerate uncomfortable internal experiences may report higher later anxiety symptoms. This finding is not surprising in itself, but its value lies in showing that experiential avoidance remains highly relevant even when the study context shifts from general student stress to AI-mediated academic life. In other words, AI may be a new technological context, but the psychological vulnerability process is familiar and theoretically grounded.

### 4.3 Why AI dependence showed negative indirect effects through experiential avoidance

The most unexpected result was that AI dependence was negatively associated with experiential avoidance, producing a negative time-ordered indirect association with anxiety symptoms. This should be interpreted as boundary-setting and disconfirming evidence against a simple harmful-use pathway rather than as proof that AI dependence is protective. Several *post hoc* explanations are possible, but they should be treated as hypotheses for future research rather than as mechanisms established by the present data. Recent evidence also suggests that the direction between AI dependence and mental health may be more complex than a simple AI-to-distress pathway (35–37). One possible explanation is that the AI dependence scale used here does not measure pathological technology addiction in the narrow clinical sense. It captures insecurity without AI, reassurance dependence on AI feedback, replacement anxiety, and competitive pressure. These experiences may be distress-related, but they may also identify students who are more actively engaged with AI-supported academic tasks and therefore less likely to avoid those tasks altogether.

A second possible explanation concerns the difference between reliance and avoidance. Reliance on AI support is not automatically equivalent to avoiding internal experiences. A student may rely on AI to check reasoning, improve writing, or organize information while still engaging with the academic task. By contrast, experiential avoidance refers to escaping unwanted thoughts and emotions. If AI functions as a cognitive scaffold rather than an avoidance device, higher AI dependence may not necessarily predict higher experiential avoidance. This interpretation is consistent with automation and overreliance research showing that dependence on automated systems can involve both efficient delegation and problematic misuse, depending on context, verification behavior, and human judgment (9, 10). At the same time, the very low R2 for experiential avoidance indicates that the present model explains only a small share of its variance; unmeasured factors such as academic stress, intolerance of uncertainty, perfectionism, self-efficacy, social comparison, and prior mental-health status may be more proximal antecedents of avoidance-related processes.

A third possibility concerns measurement meaning rather than a demonstrated coding artifact. Before model estimation, the research team rechecked permissible item ranges, scoring direction, response anchors, and item-total direction and found no reverse-scored items, miscoding, or anchor inversion. Thus, the negative association should not be attributed to an identified scoring error. It may instead reflect cultural or contextual interpretation of items about reassurance, replacement threat, and competition. Because the validated five-item AID instrument is unidimensional, *post hoc* subscale analyses were not performed; future item-level invariance and qualitative work should distinguish functional reliance from problematic dependence.

### 4.4 Why digital literacy did not moderate the model

The non-significant moderation effects involving digital literacy are also theoretically informative. Digital literacy was examined as a theoretically plausible but empirically unsupported moderator in this model. Digital literacy is often described as a competence that enables students to access, evaluate, create, and communicate information in digital environments (15, 16). Evidence from digital-health contexts also suggests that literacy-related capacities are associated with anxiety and depression, although such associations do not necessarily imply buffering effects in AI-specific models (38). Based on this view, it would be reasonable to expect digital literacy to buffer the association between AI dependence and anxiety-related processes. However, the present results did not support this buffering hypothesis. One reason may be that general digital literacy is too broad to capture the AI-specific competencies that matter in this model. Knowing how to use digital tools is not the same as knowing how to evaluate AI-generated feedback, detect hallucinations, regulate prompt dependence, recognize automation bias, or maintain academic agency when AI is available (9, 10, 17).

A second reason is that digital literacy may have mixed implications. Students with higher digital literacy may use AI more effectively and critically, but they may also be more exposed to AI tools, more aware of AI-related academic competition, and more sensitive to the speed at which AI reshapes expectations for performance. In this sense, digital literacy may not simply protect students from AI-related pressure. It may increase both capability and exposure. This may explain why digital literacy showed small associations in the model and did not operate as a clean moderator. The finding is consistent with the broader AI education literature, which argues that AI requires not only digital skills but also ethical, pedagogical, and critical capacities (11, 12).

A third possibility is statistical and conceptual. Moderation effects are often small, and detecting them requires precise measurement, sufficient variability, and a theoretically exact match between the moderator and the moderated pathway. The current digital literacy scale captures general digital competence, whereas the hypothesized moderation may depend on narrower constructs such as AI literacy, algorithmic awareness, prompt evaluation, uncertainty tolerance, or self-regulated learning. Future research should test these more specific moderators rather than assuming that broad digital literacy is sufficient.

### 4.5 Theoretical and practical implications

Theoretically, this study contributes to AI-in-education research by shifting attention from AI adoption to AI-related psychological meaning. Prior studies and policy reports have emphasized the opportunities and risks of AI in higher education (5, 11, 12), but the present study shows that mental-health implications cannot be inferred from AI exposure alone. Researchers should distinguish between AI use, AI literacy, AI dependence, AI reassurance seeking, and AI-driven competence threat. These constructs may have different psychological consequences. The findings also connect AI dependence research with the experiential avoidance literature, suggesting that established psychological processes remain important in new technological environments.

Practically, the findings suggest that universities should avoid two extreme messages: treating AI as a purely beneficial learning technology or treating AI dependence as equivalent to addiction. A more useful approach is to teach students how to use AI as support without making AI the primary source of reassurance, confidence, or decision validation. AI-literacy programs should therefore include not only technical skills but also critical evaluation, uncertainty tolerance, academic self-efficacy, and emotional self-regulation. Counseling centers, teaching units, and digital-learning offices could collaborate to identify students who rely on AI for reassurance rather than learning and provide support that strengthens independent problem solving. Because experiential avoidance was strongly linked to anxiety symptoms, interventions that reduce avoidance and improve psychological flexibility may be especially valuable in AI-mediated learning contexts.

### 4.6 Limitations and future research

Several limitations should be acknowledged. First, the study did not measure all focal constructs at every wave and did not assess baseline anxiety or baseline experiential avoidance. Consequently, the results cannot establish symptom change, within-person effects, or whether T2 experiential avoidance and T3 anxiety reflect stable pre-existing distress. The strong EA-ANX association may partly capture unmeasured prior distress. All findings are therefore described as time-lagged associations, and the indirect effect is a model-based indirect association rather than confirmed causal mediation. Future studies should use repeated measures and cross-lagged, random-intercept cross-lagged, or latent-growth models. Second, exclusive self-report measurement leaves possible social-desirability, recall, and response-style bias. Third, purposive recruitment at only two Chinese universities, restriction to students with prior AI experience, and the absence of AI-use-frequency strata limit representativeness and generalizability. Fourth, a population-level participation rate cannot be calculated because the available denominator represents baseline completers rather than all students exposed to the invitation; nevertheless, the class-based administration, reminders at every wave, two-point continuous-assessment incentive, retention, and duplicate exclusions are reported transparently. Fifth, the model explained only 2.5% of EA variance and Q²_predict values were small; therefore, its main robust contribution is the strong time-lagged EA-ANX association, not prediction of experiential avoidance from perceived AI dependence or digital literacy. Sixth, the unexpected negative effects involving AI dependence and digital literacy require replication with item-level invariance testing and qualitative research. Future studies should also test whether AI-specific competencies, such as prompt evaluation and critical appraisal of AI outputs, are stronger moderators than general digital literacy.

In sum, the study shows that the psychological consequences of AI dependence among university students are not reducible to a simple harmful-use narrative. Experiential avoidance emerged as the most important proximal correlate of later anxiety symptoms, whereas AI dependence and digital literacy showed more complex associations. The key implication is that AI governance in higher education should be psychologically informed: universities should promote responsible AI engagement while helping students preserve autonomy, tolerate uncertainty, and develop flexible coping strategies in AI-mediated learning environments.

## 5 Conclusion and future directions

### 5.1 Summary of main findings

This study examined time-lagged associations among perceived artificial intelligence dependence, experiential avoidance, digital literacy, and later anxiety symptoms. The most robust finding was the strong association between T2 experiential avoidance and T3 anxiety symptoms. By contrast, perceived AI dependence and digital literacy explained only 2.5% of experiential-avoidance variance, direct associations with anxiety were nonsignificant, moderation was unsupported, and Q²_predict values were small. The results therefore do not establish causal mechanisms or strong prediction; they identify a temporally ordered, model-based pattern that requires replication with baseline symptoms and repeated measures.

### 5.2 Significance and comparison with previous research

The present study responds to a key gap identified in the Introduction. Previous research on AI in higher education has mainly focused on learning opportunities, intelligent tutoring, academic writing, ethics, and institutional governance (5, 11, 12), whereas fewer studies have examined AI dependence as a psychological construct linked to student mental health. By applying the recently developed AI dependence scale among university students (14), this study moves beyond the question of whether students use AI and instead asks whether perceived reliance on AI support is connected with later anxiety-related processes. This distinction is important because frequent AI use may reflect productive academic assistance, whereas psychological dependence involves insecurity, reassurance seeking, and perceived competence threat.

The findings also extend research on digital literacy. Existing work frames digital literacy as a multidimensional competence that allows students to evaluate information, communicate, create content, and act critically in digital environments (15, 16). The current results did not show significant moderation by digital literacy, which differs from the expectation that digital literacy would necessarily buffer the psychological consequences of AI dependence. One possible explanation is that digital literacy may not operate as a simple protective factor. Students with higher digital literacy may be more capable of using AI critically, but they may also be more exposed to AI tools, more aware of AI-related competence pressures, or more strategic in using AI for academic work. Thus, digital literacy may shape AI use in complex ways that are not fully captured by a single interaction term.

The most robust result was the strong association between experiential avoidance and anxiety symptoms. This aligns with the acceptance and commitment therapy literature, in which experiential avoidance and psychological inflexibility are viewed as transdiagnostic processes related to anxiety and emotional distress (18, 21, 22). Compared with prior cross-sectional work, the advantage of the present study is its three-wave structure: the predictor/moderator, putative explanatory process, and outcome were measured at separate time points. This design improves the temporal ordering of the model and reduces same-time measurement bias, although it still does not establish strict causality. In this sense, the study contributes a more cautious but methodologically stronger account of how AI-related psychological dependence may be situated within broader anxiety-related processes.

### 5.3 Interpretation of unexpected findings and study limitations

The negative paths from AI dependence and digital literacy to experiential avoidance were unexpected and should not be interpreted as evidence of a protective mechanism. The pre-estimation scoring audit confirmed permissible response ranges, consistent response anchors, and the intended item direction; no reverse-scored items, miscoding, or anchor inversion was identified. Because the validated five-item AID instrument is unidimensional, *post hoc* subscale analyses were not performed. The negative association may instead reflect contextual differences between functional AI-supported engagement and problematic reassurance dependence, a distinction that should be tested directly in future studies.

The non-significant moderation effects suggest that digital literacy may not be sufficient by itself to alter the relationship between AI dependence, experiential avoidance, and anxiety. Digital literacy may need to be separated into more specific dimensions, such as technical competence, information evaluation, AI-output verification, ethical awareness, and self-regulated learning. A global digital literacy score may mask these distinct pathways. In addition, the relatively low explained variance in experiential avoidance indicates that other psychological or contextual factors may be more proximal predictors of avoidance, such as academic stress, intolerance of uncertainty, self-efficacy, perfectionism, social comparison, or prior mental-health status.

Several limitations should be acknowledged. First, although the three-wave design establishes a basic temporal order, the study did not repeatedly measure AI dependence, experiential avoidance, digital literacy, and anxiety symptoms at every wave. As a result, the findings should be interpreted as time-lagged associations rather than strict causal or within-person change effects. Second, the study relied on self-report questionnaires, which may be affected by recall bias, social desirability, and response-style bias. Third, purposive online sampling of university students with AI-use experience limits the generalizability of the findings. Fourth, although wave-specific records indicated very low attrition and only two duplicate-record exclusions, purposive online sampling limits formal evaluation of nonresponse and selection bias; future reporting should continue to document eligibility, retention, exclusion, and analytic-sample construction (39). Fifth, although the supplementary covariate-adjusted sensitivity analysis indicated that the focal path pattern was stable, future work should provide preregistered covariate specifications, richer attrition diagnostics, and additional robustness checks using alternative estimators such as covariance-based SEM or longitudinal panel models. These limitations do not negate the findings, but they require cautious interpretation and stronger designs in subsequent research.

### 5.4 Future directions and practical implications

Future research should build on these findings in several ways. Longitudinal studies should measure all core constructs at each wave and use cross-lagged panel, latent growth, or random-intercept models to distinguish between stable between-person differences and within-person change. Experimental or intervention studies could test whether AI-literacy training, uncertainty-tolerance training, or acceptance-based psychological interventions reduce maladaptive reliance on AI reassurance and later anxiety symptoms. Future work should also decompose digital literacy into more specific AI-related competencies, including prompt evaluation, output verification, algorithmic awareness, ethical reasoning, and self-regulated learning. Such work would clarify whether particular dimensions of digital or AI literacy protect students from psychological overreliance more effectively than global digital literacy.

In practical terms, the findings suggest that universities should not frame AI use only as a technical or academic-integrity issue. AI support is now part of students’ learning ecology, and responsible AI education should include psychological and behavioral components, particularly as AI is increasingly discussed as both a public-health education resource and a mental-health care challenge (6, 7). Universities can help students use AI as a learning aid without making AI the primary source of reassurance, self-worth, or academic confidence. Mental-health services and teaching units could collaborate to develop AI-literacy programs that combine technical skill, critical evaluation, academic self-efficacy, and emotional self-regulation. Because experiential avoidance was strongly associated with anxiety symptoms, interventions that help students tolerate uncertainty, engage with difficult tasks, and reduce avoidance may be especially useful in AI-mediated learning environments.

Overall, this study contributes to the emerging public-health discussion on AI in higher education by showing that the psychological meaning of AI dependence matters. AI dependence should not be equated automatically with technology use frequency or addiction. Rather, it should be examined in relation to students’ emotional responses, coping patterns, digital competence, and mental-health outcomes. By linking AI dependence, experiential avoidance, digital literacy, and anxiety symptoms in a time-lagged framework, the study may inform psychologically sensitive AI-literacy and student-support initiatives that encourage productive AI engagement while also protecting students’ autonomy and well-being.

## Data Availability

The original contributions presented in the study are included in the article/**Supplementary Material**, further inquiries can be directed to the corresponding author/s.

## References

[1] Li W Zhao Z Chen D Peng Y Lu Z . Prevalence and associated factors of depression and anxiety symptoms among college students: a systematic review and meta-analysis. J Child Psychol Psychiatry. (2022) 63:1222–30. doi: 10.1111/jcpp.13606 35297041

[2] Duraku ZH Davis H Hamiti E . Mental health, study skills, social support, and barriers to seeking psychological help among university students: a call for mental health support in higher education. Front Public Health. (2023) 11:1220614. doi: 10.3389/fpubh.2023.1220614 37920583 PMC10619655

[3] Auerbach RP Mortier P Bruffaerts R Alonso J Benjet C Cuijpers P . WHO World Mental Health Surveys International College Student Project: prevalence and distribution of mental disorders. J Abnorm Psychol. (2018) 127:623–38. doi: 10.1037/abn0000362 30211576 PMC6193834

[4] Lipson SK Zhou S Abelson S Heinze J Jirsa M Morigney J . Trends in college student mental health and help-seeking by race/ethnicity: findings from the national healthy minds study, 2013-2021. J Affect Disord. (2022) 306:138–47. doi: 10.1016/j.jad.2022.03.038 35307411 PMC8995361

[5] Zawacki-Richter O Marin VI Bond M Gouverneur F . Systematic review of research on artificial intelligence applications in higher education: where are the educators? Int J Educ Technol High Educ. (2019) 16:39. doi: 10.1186/s41239-019-0171-038164791

[6] Wang J Li J . Artificial intelligence empowering public health education: prospects and challenges. Front Public Health. (2024) 12:1389026. doi: 10.3389/fpubh.2024.1389026 39022411 PMC11252473

[7] Simoes JP ten Klooster P Neff PK Niemann U Kraiss J . Editorial: Artificial intelligence and mental health care. Front Public Health. (2024) 12:1461446. doi: 10.3389/fpubh.2024.1461446 39139670 PMC11319249

[8] Vanden Abeele MMP . Digital wellbeing as a dynamic construct. Commun Theory. (2021) 31:932–55. doi: 10.1093/ct/qtaa024

[9] Parasuraman R Riley V . Humans and automation: use, misuse, disuse, abuse. Hum Factors. (1997) 39:230–53. doi: 10.1518/001872097778543886

[10] Lyell D Coiera E . Automation bias and verification complexity: a systematic review. J Am Med Inform Assoc. (2017) 24:423–31. doi: 10.1093/jamia/ocw105 27516495 PMC7651899

[11] Miao F Holmes W . Guidance for Generative AI in Education and Research. Paris: UNESCO (2023). Available online at: https://www.unesco.org/en/articles/guidance-generative-ai-education-and-research (Accessed October 14, 2026).

[12] Kasneci E Sessler K Kuchemann S Bannert M Dementieva D Fischer F . ChatGPT for good? On opportunities and challenges of large language models for education. Learn Individ Differ. (2023) 103:102274. doi: 10.1016/j.lindif.2023.10227442574925

[13] Chan CKY Hu W . Students' voices on generative AI: perceptions, benefits, and challenges in higher education. Int J Educ Technol High Educ. (2023) 20:43. doi: 10.1186/s41239-023-00411-838164791

[14] Morales-Garcia WC Sairitupa-Sanchez LZ Morales-Garcia SB Morales-Garcia M . Development and validation of a scale for dependence on artificial intelligence in university students. Front Educ. (2024) 9:1323898. doi: 10.3389/feduc.2024.1323898

[15] Ng W . Can we teach digital natives digital literacy? Comput Educ. (2012) 59:1065–78. doi: 10.1016/j.compedu.2012.04.01642574925

[16] Ustundag MT Gunes E Bahcivan E . Turkish adaptation of digital literacy scale and investigating pre-service science teachers digital literacy. J Educ Future. (2017) 16:19–29.

[17] Long D Magerko B . (2020). “ What is AI literacy? Competencies and design considerations”, in: Proceedings of the 2020 CHI Conference on Human Factors in Computing Systems (New York: Association for Computing Machinery), 1–16. doi: 10.1145/3313831.3376727

[18] Bond FW Hayes SC Baer RA Carpenter KM Guenole N Orcutt HK . Preliminary psychometric properties of the Acceptance and Action Questionnaire-II: a revised measure of psychological inflexibility and experiential avoidance. Behav Ther. (2011) 42:676–88. doi: 10.1016/j.beth.2011.03.007 22035996

[19] Hayes SC Luoma JB Bond FW Masuda A Lillis J . Acceptance and commitment therapy: model, processes and outcomes. Behav Res Ther. (2006) 44:1–25. doi: 10.1016/j.brat.2005.06.006 16300724

[20] Kashdan TB Rottenberg J . Psychological flexibility as a fundamental aspect of health. Clin Psychol Rev. (2010) 30:865–78. doi: 10.1016/j.cpr.2010.03.001 21151705 PMC2998793

[21] Tyndall I Waldeck D Pancani L Whelan R Roche B Dawson DL . The Acceptance and Action Questionnaire-II as a measure of experiential avoidance: concerns over discriminant validity. J Contextual Behav Sci. (2019) 12:278–84. doi: 10.1016/j.jcbs.2018.09.00542574925

[22] Angelakis I Panagioti M Austin JL Gooding P . Experiential avoidance in depression, anxiety, obsessive-compulsive related, and posttraumatic stress disorders: a comprehensive systematic review and meta-analysis. J Contextual Behav Sci. (2022) 24:65–78. doi: 10.1016/j.jcbs.2022.03.00742574925

[23] Chawla N Ostafin B . Experiential avoidance as a functional dimensional approach to psychopathology: an empirical review. J Clin Psychol. (2007) 63:871–90. doi: 10.1002/jclp.20400 17674402

[24] Spitzer RL Kroenke K Williams JWB Lowe B . A brief measure for assessing generalized anxiety disorder: the GAD-7. Arch Intern Med. (2006) 166:1092–7. doi: 10.1001/archinte.166.10.1092 16717171

[25] Lowe B Decker O Muller S Brahler E Schellberg D Herzog W . Validation and standardization of the Generalized Anxiety Disorder Screener (GAD-7) in the general population. Med Care. (2008) 46:266–74. doi: 10.1097/MLR.0b013e318160d093 18388841

[26] World Medical Association . World Medical Association Declaration of Helsinki: ethical principles for medical research involving human participants. JAMA. (2025) 333:71–4. doi: 10.1001/jama.2024.21972 39425955

[27] Kock N . Common method bias in PLS-SEM: a full collinearity assessment approach. Int J e-Collab. (2015) 11:1–10. doi: 10.4018/ijec.2015100101

[28] Hair JF Hult GTM Ringle CM Sarstedt M . A Primer on Partial Least Squares Structural Equation Modeling (Pls-Sem). 3rd ed. Thousand Oaks (CA): Sage (2022).

[29] Ringle CM Wende S Becker JM . Smartpls 4. Bönningstedt: SmartPLS (2024). Available online at: https://www.smartpls.com (Accessed October 14, 2026).

[30] Fornell C Larcker DF . Evaluating structural equation models with unobservable variables and measurement error. J Mark Res. (1981) 18:39–50. doi: 10.1177/002224378101800104

[31] Dijkstra TK Henseler J . Consistent partial least squares path modeling. MIS Q. (2015) 39:297–316. doi: 10.25300/MISQ/2015/39.2.02

[32] Henseler J Ringle CM Sarstedt M . A new criterion for assessing discriminant validity in variance-based structural equation modeling. J Acad Mark Sci. (2015) 43:115–35. doi: 10.1007/s11747-014-0403-830311153

[33] Shmueli G Sarstedt M Hair JF Cheah JH Ting H Vaithilingam S . Predictive model assessment in PLS-SEM: guidelines for using PLSpredict. Eur J Mark. (2019) 53:2322–47. doi: 10.1108/EJM-02-2019-018935579975

[34] Podsakoff PM MacKenzie SB Lee JY Podsakoff NP . Common method biases in behavioral research: a critical review of the literature and recommended remedies. J Appl Psychol. (2003) 88:879–903. doi: 10.1037/0021-9010.88.5.879 14516251

[35] Huang S Lai X Ke L Li Y Wang H Zhao X . AI technology panic-is AI dependence bad for mental health? A cross-lagged panel model and the mediating roles of motivations for AI use among adolescents. Psychol Res Behav Manag. (2024) 17:1087–102. doi: 10.2147/PRBM.S440889 38495087 PMC10944174

[36] Fu Y Ren F Lin J . Apriori algorithm based prediction of students mental health risks in the context of artificial intelligence. Front Public Health. (2025) 13:1533934. doi: 10.3389/fpubh.2025.1533934 40013053 PMC11860887

[37] Lai L Pan Y Xu R Jiang Y . Depression and the use of conversational AI for companionship among college students: the mediating role of loneliness and the moderating effects of gender and mind perception. Front Public Health. (2025) 13:1580826. doi: 10.3389/fpubh.2025.1580826 40520273 PMC12162938

[38] Akingbade O Adeleye K Fadodun OA Fawole IO Li J Choi EPH . eHealth literacy was associated with anxiety and depression during the COVID-19 pandemic in Nigeria: a cross-sectional study. Front Public Health. (2023) 11:1194908. doi: 10.3389/fpubh.2023.1194908 37427252 PMC10323132

[39] von Elm E Altman DG Egger M Pocock SJ Gotzsche PC Vandenbroucke JP . The Strengthening the Reporting of Observational Studies in Epidemiology (STROBE) statement: guidelines for reporting observational studies. PloS Med. (2007) 4:e296. doi: 10.1371/journal.pmed.0040296 17941714 PMC2020495

