# Supplementary material for: Perceived artificial intelligence dependence and anxiety symptoms among university students: a three-wave study of experiential avoidance and digital literacy
Source: Front Psychiatry. 2026 Oct 19;17:1934192. doi: 10.3389/fpsyt.2026.1934192 (PMC13724574; doi:10.3389/fpsyt.2026.1934192)
Supplement: Supplementary file 1 [file Table1.docx]

**Supplementary Table S1. Unadjusted and covariate-adjusted sensitivity estimates**

Sensitivity analyses were conducted using standardized composite-score regressions based on scale mean scores. The adjusted model included gender, age, family monthly income, maternal education years, and paternal education years as covariates predicting experiential avoidance and anxiety symptoms.

| **Path** | ***Unadjusted beta*** | ***Unadjusted p*** | ***Adjusted beta*** | ***Adjusted p*** |
| --- | --- | --- | --- | --- |
| AID -> ANX | -.029 | .134 | -.029 | .139 |
| AID -> EA | -.105 | <.001 | -.111 | <.001 |
| DL -> ANX | -.010 | .619 | -.016 | .421 |
| DL -> EA | -.094 | <.001 | -.089 | <.001 |
| DL x AID -> ANX | .019 | .286 | .019 | .290 |
| DL x AID -> EA | -.018 | .462 | -.018 | .455 |
| DL x EA -> ANX | -.002 | .916 | -.003 | .832 |
| EA -> ANX | .676 | <.001 | .669 | <.001 |
| AID -> EA -> ANX | -.071 | NA | -.074 | NA |

Note. beta values are standardized estimates. The adjusted model controlled for gender, age, family monthly income, maternal education years, and paternal education years. The indirect association was calculated as the product of the AID -> EA and EA -> ANX component paths; a separate p value is not reported for the product term in this sensitivity table. AID, artificial intelligence dependence; EA, experiential avoidance; ANX, anxiety symptoms; DL, digital literacy; NA, not applicable.
